# Enhancing *Escherichia coli* production of material proteins using circular mRNAs

**DOI:** 10.1128/aem.01579-25

**Published:** 2025-10-07

**Authors:** Alden Filko, Fuzhong Zhang

**Affiliations:** 1 Department of Energy, Environmental and Chemical Engineering, Washington University in St. Louis 685423, St. Louis, Missouri, USA; 2 Division of Biology & Biomedical Sciences, Washington University in St. Louis 318396 , St. Louis, Missouri, USA; 3 Institute of Materials Science & Engineering, Washington University in St. Louis 630240, St. Louis, Missouri, USA; University of Illinois Urbana-Champaign, Urbana, Illinois, USA

**Keywords:** biomaterials, circular mRNA, protein expression

## Abstract

Industrial bioproduction of proteins, particularly protein-based materials (PBMs) like spider silk and elastin proteins, is rapidly expanding. PBMs often have high molecular weights and are highly repetitive, transcribed from long and repetitive mRNAs that are prone to degradation in microbial hosts. As a result, recombinant expression of PBMs often has low protein yields. In this study, we engineered a circular mRNA expression system to enhance mRNA stability and protein expression. The system uses self-cleaving ribozymes to form circular mRNA structures and a pair of insulation RNA loops to improve protein translation. When tested using a green fluorescent protein (GFP) reporter, the engineered circular mRNA enhanced GFP expression by 1.5-fold compared to expression from a linear construct. mRNA circularization was further confirmed using reverse transcription followed by DNA amplification and sequencing. We also demonstrate the effectiveness of circular mRNA in enhancing the expression of various material proteins, including a 96-mer repeat of *Nephila clavipes* dragline silk, a titin repeat, a mussel foot protein oligomer, and an silk-amyloid repeat, resulting in up to 2.5-fold increase in protein yield. Additionally, the circular mRNA system also improved the stability of the PBM-encoding plasmid. Overall, the circular RNA expression system enhances both the expression level and plasmid stability and is suitable for various protein production applications.

**IMPORTANCE:**

Industrial bioproduction of complex proteins is limited by unstable expression. Long and repetitive proteins have unstable expression and often yield truncated products that will change the properties of the final materials. We show that by using a self-circularizing mRNA system, the expression is stabilized to not only increase yields but also prevent truncated products. The ability to produce full-length proteins consistently and control their size offers precise control over protein properties, making it highly relevant for products with specific mechanical properties. The study showcases the potential for scaling up protein production in industrial bioreactors under challenging conditions. The findings contribute to synthetic biology tools and offer new avenues for manufacturing bioproducts at an industrial scale.

## INTRODUCTION

The microbial production of recombinant proteins has been widely used in industry for producing various products such as antibodies, therapeutic proteins, and industrial enzymes (1–3). In addition to these traditional uses, there is growing interest in employing microbially produced proteins to develop renewable and biodegradable materials, often referred to as protein-based materials (PBMs) (4, 5). Examples of PBMs include materials like spider silk, elastin, sucker ring teeth proteins, mussel foot proteins, and titin (6–10). Many of these PBMs exhibit superior mechanical properties compared to conventional polymeric materials. This makes them highly suitable for mechanically demanding applications (11–13). Furthermore, PBMs are typically biocompatible and bioabsorbable, which extends their usability into various biomedical applications (8, 14–17). Unlike many water-soluble therapeutic proteins and enzymes with well-defined folded structures, PBMs are often disordered and insoluble when expressed in microbial hosts. This presents unique challenges in their microbial production. The low solubility of PBMs usually requires their purification to be performed in urea or guanidine buffer to avoid protein aggregation. Moreover, many PBMs have highly repetitive sequences, biased amino acid compositions, and large molecular weights (MWs) (18). These features can significantly complicate their expression at high levels and high yields.

Despite these challenges, recent advances in synthetic biology have led to the development of multiple strategies to overcome the difficulties in high-yield PBM production (18). These approaches include extensive optimization of PBM codon usage to reduce nucleic acid sequence repetitiveness and post-translational protein ligation techniques to produce high MW PBMs (9, 19). Producing PBMs with high titer and yield is critical for their practical applications.

An often-overlooked aspect of PBM production is the stability of their mRNAs, which directly impacts the expression levels of PBMs (20). Generally, microbial mRNAs are short-lived, with the lifespans of most native mRNAs in *Escherichia coli* ranging from 1 to 6 min (21). The high MW and highly repetitive PBM protein sequences are translated from long mRNAs, which are particularly susceptible to degradation by native ribonucleases (RNases), leading to even shorter lifetimes and consequently affecting protein yield. The primary pathway for mRNA degradation involves RNase R, RNase II, and PNPase (22). These RNases predominantly target the 3′ end of mRNA, progressively shortening it by cleaving off bases. Although deletion of these RNases can lead to an increased mRNA lifetime, doing so also hinders cell growth due to the disruption of normal mRNA degradation processes (22). One strategy to improve mRNA stability involves forming circular mRNA. Two independent research groups have explored using stop-codon-free circular mRNA to express recombinant proteins, including spider silk (23, 24). These methods employed ribozymes to circularize mRNAs that encode a single repeat of the spider silk protein. This circular configuration allows ribosomes to translate repeatedly for multiple rounds on the circular mRNA before dissociating, thus facilitating the production of proteins with numerous repeat units. However, the absence of a stop codon causes random ribosome disassociation from the mRNA, resulting in protein products with varying molecular weights (23, 24). This variability limits applications that require uniform protein characteristics. Notably, circularization also protects mRNA from RNase degradation by eliminating the free 5′ and 3′ ends where many RNase degradation starts (22, 25), thus potentially enhancing mRNA stability and protein yield. Despite these benefits, the explicit impact of circular mRNA on the overall yield of PBMs has not yet been fully explored, highlighting a research opportunity in synthetic biology and protein engineering.

In this study, we aimed to develop circular mRNA that includes a stop codon to enable the production of proteins with defined MWs and sequences and to evaluate its effectiveness in enhancing the yield of various PBMs. By applying this method to *E. coli*-based production systems, we observed that mRNA circularization improves the expression of a green fluorescent protein (GFP) by 1.5-fold. This strategy was subsequently applied to four different material proteins, including recombinant titin, Mfp repeats, silk-amyloid repeats, and recombinant spider silk, encompassing diverse sizes and repetitive sequences. Circular mRNA substantially increased the yields of multiple PBMs by up to 2.5-fold. This significant improvement underscores the potential of mRNA circularization to overcome the expression problem of repetitive proteins, providing a powerful tool for optimizing the microbial production of various PBMs.

## RESULTS

### Design and construction of a circular mRNA expression system

**Fig 1:**
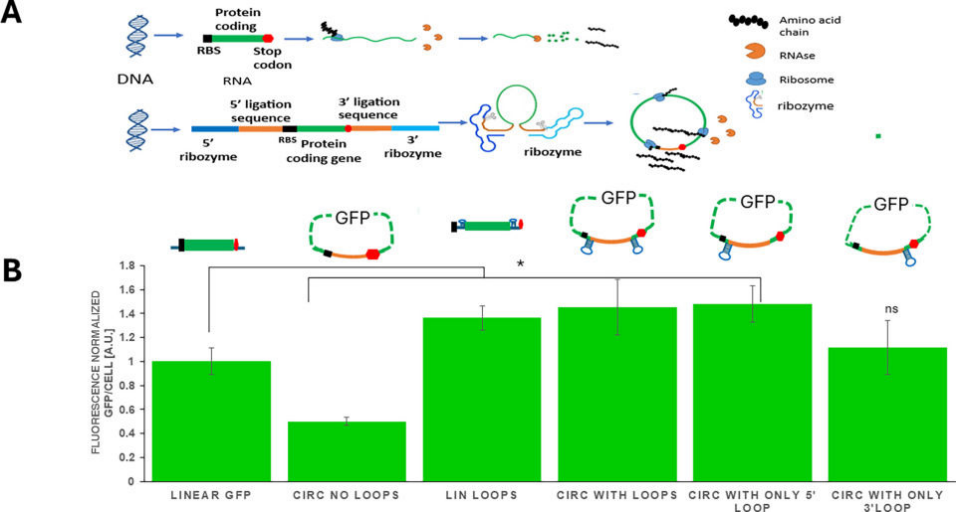
Circular mRNA improves GFP expression. (**A**) Protein translation from circular mRNA. Protein expression from a standard linear mRNA. mRNA degradation by RNases reduces mRNA lifetime and protein yields. Protein expression from a circular RNA. Due to the lack of free 5′ and 3′ ends, circular mRNA is more stable, leading to enhanced protein expression level. (**B**) GFP fluorescence per cell normalized to linear mRNA expression for each mRNA construct. Error bars represent standard deviation from biological replicates (*n* = 3). *, *P* < 0.05; ns, *P* > 0.05, unpaired *t*-test compared to linear GFP.

Compared to linear mRNA, circular RNAs exhibit heightened stability due to their absence of free 5′ and 3′ ends, typically the recognition sites of exonucleases for mRNA degradation (Fig. 1A) (26). Circular RNA can be made in several ways and has been extensively studied in eukaryotic cells related to vaccine applications (27). Eukaryotes have a more advanced system for mRNA regulation, largely due to the added complexity of mRNA modification and mRNA-protein interaction (28). The addition of 5′ caps and poly-A tails enables multiple stabilization pathways, not only through covalent circularization but also via protein-mediated circularization (28). A typical mRNA covalent circularization strategy involves the forward or back splicing of exons, which is impractical in a bacterial host (27, 29). Methods for bacterial mRNA circularization, such as the T4 phage *td* intron or the Tornado system, have been developed (23, 26). Comparing these two methods, the Tornado system produced substantially higher levels of circular mRNA than the *td* intron system; thus, it is preferred (30). The Tornado system involves self-cleaving ribozymes and leverages the native RtcB ligase in *E. coli* (26). This system expresses a coding mRNA flanked by two ribozymes at both 5′ and 3′ ends of the coding mRNA. Upon transcription, the ribozymes undergo self-cleavage, producing new 5′ and 3′ ends, which *E. coli*’s native RtcB ligase can recognize to generate a circular mRNA. We adopted the Tornado system by choosing a pair of ribozymes with fast and robust folding kinetics (approximately 10 min^−1^), much shorter than the typical half-life of an mRNA (21, 30–32). Thus, this strategy can efficiently generate the sticky ends for ligation before mRNA degradation. Within the circularized mRNA, we kept all essential elements for protein translation, including the ribosome binding site (RBS), coding region, and the stop codon. Therefore, a complete protein with fully controlled and uniform MW can be translated (Fig. 1A) (23, 24). One concern with the circular mRNA was the effect of RNA secondary structures on ribozyme function. Furthermore, the post-ligation region may interact with the nearby RBS, thus affecting protein translation. To address these concerns, we added a hairpin loop between each ribozyme and coding region inside the circular mRNA to insulate the translated region (Fig. 1; Fig. S1) (32).

### Testing the effects of circular mRNA using GFP

We first used GFP to evaluate the effect of circular mRNA on protein production, taking advantage of GFP’s ease of quantification. To compare the impact of circular mRNA, we also constructed RNAs without the insulation loops or the ribozymes (Fig. 1B). Plasmids capable of transcribing these RNAs were transformed into *E. coli* strain followed by cultivation in Luria-Bertani (LB) media. GFP levels were quantified via total fluorescence using a fluorescent plate reader. The results revealed a notable increase in GFP production when using the circular mRNA containing the insulation loops. The final GFP yield was 1.5× higher than that of a linear RNA with an identical coding sequence (Fig. 1B). We noticed that the circular mRNA without the insulating loops showed decreased GFP expression, suggesting the loop effectively prevented undesirable secondary structures on the mRNA (27, 33). To determine which insulating loop played the dominant role, we further generated circular mRNA constructs containing a single loop placed either at the 5′ or 3′ end of the GFP coding region. GFP expression from these constructs revealed that circular mRNA with only the 5′ loop produced GFP levels similar to constructs with both loops, whereas circular mRNA with only the 3′ loop showed reduced expression (Fig. 1B). These observations suggest that circular mRNA with a 5′ insulation loop can effectively improve protein expression.

### Confirmation of mRNA circularization

**Fig 2:**
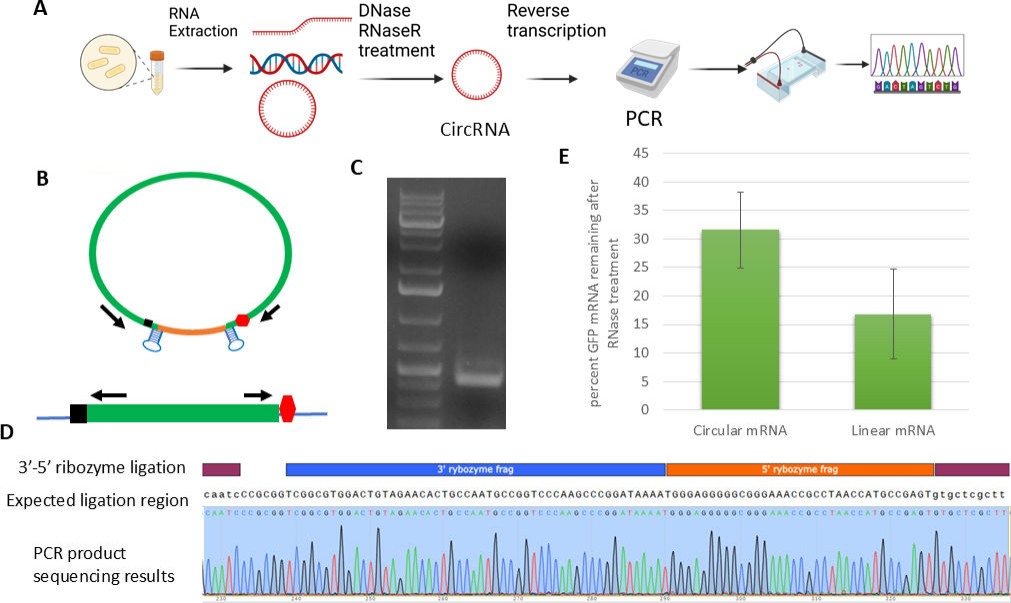
Confirmation of circular mRNA. (**A**) Workflow of RNA extraction, yielding linear RNA, DNA, and circular RNA is shown, followed by treatment to remove linear RNA and all DNA, reverse transcription, and circular RNA detection. (**B**) PCR primers that annealed at either end of GFP cDNA were used to amplify the ligation region. (**C**) Electrophoresis confirmed the successful amplification of the ligation region. (**D**) Sanger sequencing result of the PCR-amplified DNA band from panel C. (**E**) Percentage of total GFP mRNA remaining after Rnase R linear RNA digestion. qPCR primers targeting center of GFP and random primers used for reverse transcription.

We performed RNA analysis to confirm that the GFP mRNAs were circularized (Fig. 2A). First, total nucleic acids were extracted from cells carrying the circular mRNA constructs. The circular RNA was then enriched by digesting the pool of extracted total nucleic acids with DNase and RNase R, which degrade plasmid DNAs and linear RNAs. The remaining RNA was then reverse transcribed using random primers. Next, primers specifically targeting the ends of the GFP sequence but converging on the ligation region were designed (Fig. 2B). This design is critical because it only amplifies circular GFP mRNA but not linear mRNA. Polymerase chain reaction (PCR) using these primers yielded a pronounced band (Fig. 2C), indicating the formation of circular mRNA. The PCR band was subsequently extracted from the gel and subjected to Sanger sequencing to confirm its sequence further. The sequencing results showed that the 3′ untranslated region (UTR) of GFP was ligated with its 5′ UTR, containing the designed RBS (Fig. 2D). Thus, our results confirmed that the designed construct had successfully formed circular mRNA. To further assess the mRNA circularization efficiency, we calculated the fraction of GFP-encoding mRNA resistant to RNase R digestion (see Methods). Under the vendor-recommended conditions, 17% of the GFP-coding mRNA from the linear construct remained undigested, whereas 32% of the RNA from the circular construct was resistant to digestion, corresponding to an estimated circularization efficiency of at least 18% (Fig. 2E).

### Circular expression of long repetitive proteins

After confirming the enhanced GFP expression, we aimed to determine whether the engineered circular mRNA can improve the expression of PBMs. We selected various material proteins known for their advantageous mechanical properties and potential applications, covering a range of sizes, properties, and uses. (i) 12× titin: a 3× repeat of the titin immunoglobulin (Ig) domains 67–70 (containing a total of 12 Ig domains). With a MW of 127 kDa, this protein can form fibers with high toughness and damping energy due to its reversible unfolding (8). (ii) Mfp5^(3)^: a 28 kDa disordered protein containing three repeats of mussel foot protein 5 (7). Mfp5^(3)^ exhibits a robust underwater adhesion to a wide range of surface types. Its adhesion force is 5.7-fold higher than recombinant Mfp5 from mussels, making it an attractive candidate for various repair applications in wet and aqueous environments (12). (iii) 96× FGA: a 284 kDa silk-amyloid hybrid protein containing 96 repeats of the FGAILSS amyloid flanked by flexible protein linkers from spider silk. Fibers of 96× FGAILSS have high tensile strength and toughness, comparable to natural spider silk (13). (iv) 96× Spidroin: a 277 kDa recombinant spider silk protein containing 96 repeating units of a dragline silk protein MaSp1 from *Nephila clavipes* (10). The *Nephila clavipes* dragline silk is famous for its unique combination of extraordinarily high strength and elasticity, but notorious for its difficulty in recombinant expression.

**Fig 3:**
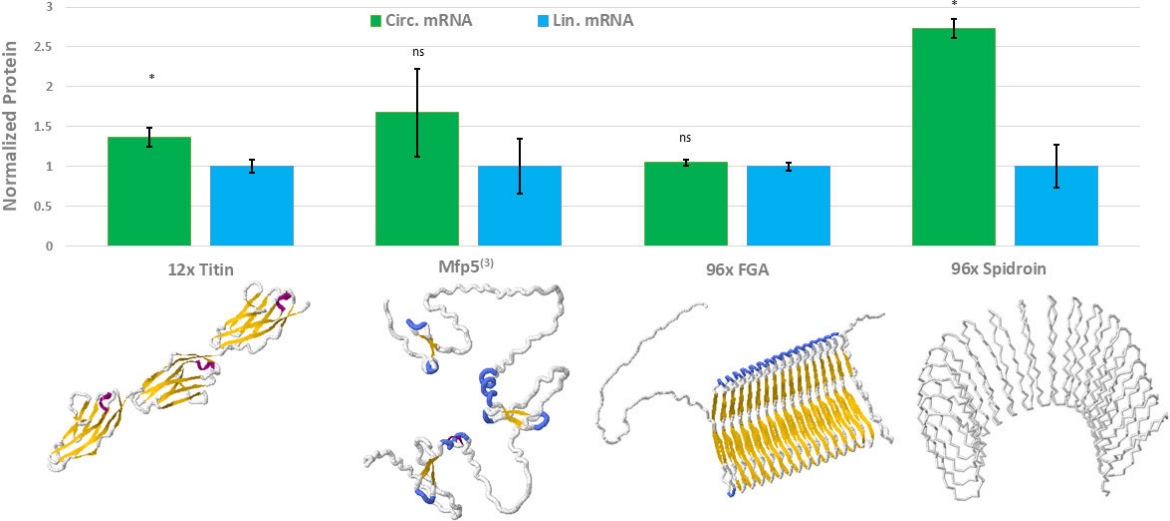
Expression of repetitive material protein using circular mRNA. Protein expression levels were measured by quantifying the target protein band intensity from SDS-PAGE gels. Error bars represent standard deviation from biological replicates (*n* = 3). *, *P* < 0.05; ns, *P* > 0.05, unpaired *t*-test compared to linear mRNA expression. Protein structures were predicted using AlphaFold2 (34). The 96× spider silk structure shown represents a 20× repeat for size restrictions. PDB files used: titin ig: 2rjm, others predicted with AlphaFold2.

Synthetic DNA coding for these PBMs was cloned into the same expression vectors with or without the circular mRNA design (containing both insulating loops). Protein expression levels were measured using quantitative SDS-PAGE. Statistically significant improvements in protein expression were observed for both 12× titin and 96× Spidroin, with a notable 2.5-fold enhancement in the latter case (Fig. 3; Fig. S2). The lack of improvement in 96× FGA expression remains unclear but may be due to a reduced mRNA circularization efficiency caused by its specific sequence. Additionally, we measured the growth rates of cells expressing these proteins from either linear or circular mRNAs and found minimal differences between these constructs (Fig. S3).

Furthermore, we evaluated whether the circular mRNA construct could provide more stable transcript levels compared to the linear construct across different batches of cultures. Five colonies of each circular and linear 96× Spidroin construct were cultivated in parallel, and total mRNA was extracted during both exponential and stationary growth phases. Transcript levels of 96× Spidroin were quantified using quantitative reverse transcription-PCR (RT-qPCR). The linear mRNA construct exhibited substantial variability in transcript abundance between cultures (coefficient of variance [CV] = 1.42, Fig. S4). In contrast, the circular mRNA construct showed more consistent transcript levels across all replicates (CV = 0.63).

We hypothesized that the batch-to-batch expression stability observed with circular mRNA originates from improved plasmid stability. When high MW PBMs, such as 96× Spidroin, are expressed from plasmids using linear mRNA, rapid mRNA degradation and ongoing transcription can create a futile cycle. This cycle imposes a significant metabolic burden on the host cell, often leading to genetic mutations in the PBM-encoding plasmid. Consequently, previous efforts to express high MW Spidroin required extensive screening to identify unmutated plasmids—a laborious process (10). To assess the genetic stability of the 96× Spidroin plasmid, we cultured multiple colonies from the same agar plate overnight, performed minipreps, and sequenced the plasmids. All five colonies tested carried mutated plasmids (Fig. S5). In contrast, when using the circular mRNA construct encoding the same 96× Spidroin sequence, we observed significantly improved plasmid stability: three out of five plasmids maintained the correct sequence. These results demonstrate that circular mRNA improves plasmid stability compared to linear mRNA. This underscores the potential of the circular construct in enhancing both the yield and stability of challenging protein productions.

## DISCUSSION

In this paper, we have developed an expression system that leverages self-circularizing mRNA to improve PBM expression. The designed circular mRNA expression systems effectively enhanced the production of multiple material proteins, including 12× titin, Mfp5^(3)^, 96-FGA, and 96× Spidroin, spanning a wide MW range from 27 to 284 kDa and diverse sequence features. Among these proteins, the most substantial improvement was observed in producing the 96× spider silk protein. This increase is attributed, in part, to the enhanced stability of the circular mRNA form. Codon usage has been shown to affect the translational rate, affecting mRNA stability (20, 35). Due to the repetitive use of codons, repetitive coding sequences slow down the translation, rendering mRNA prone to degradation and decreasing its stability. Circular mRNA stabilizes mRNA due to the absence of free 5′ and 3′ ends, thus improving protein production.

Unlike previous circular mRNA methods that produced various protein products with different MWs due to the absence of a stop codon, our approach effectively resolved this problem. It consistently produced uniform protein products with defined MW. This is crucial for some material applications because protein MW often affects the properties of the resulting materials. For example, in the case of synthetic silk fibers, the fiber’s ultimate strength and toughness strongly depend on protein MW (36). Thus, our circular mRNA design offers more precise control over the fiber’s mechanical properties than previous circular mRNA designs (23).

Furthermore, our circular mRNA construct appears to enhance the production of full-length 96× Spidroin protein, in contrast to the linear mRNA construct, which predominantly yields truncated fragments (Fig. S2). This is crucial because protein fragments resulting from partially degraded mRNAs share similar physical properties with the full-length proteins and are difficult to separate. Their co-production presents significant hurdles for PBM applications. Our circular mRNA strategy effectively reduced this problem. Additionally, the expression of 96× Spidroin protein from circular mRNA substantially improved the stability of the PBM-encoding plasmid, saving time needed for extensive screening of unmutated plasmid when using linear mRNA. The increased consistency in protein expression is especially applicable in industrial bioproduction, where failed batches can substantially increase production costs and hinder profits if expression is lost and yields are low.

Overall, this study demonstrated significant advancements in using circular mRNA to promote protein production, particularly for applications requiring the expression of uniform and repetitive proteins. This method stabilizes protein output and enhances the overall yield by circumventing the common problem in expressing repetitive proteins from linear mRNA. Future work on optimizing protein secretion and solubility may be necessary to further enhance the protein production process. The robustness of circular mRNA also provides a reliable framework for other large or complex proteins, potentially useful for bacterial expression of eukaryotic proteins with short-lived mRNAs. Additionally, this technology can be used in combination with other synthetic biology tools (34, 37), enabling more stable and efficient synthesis of complex proteins and pathways for various new applications, potentially opening new avenues for manufacturing bioproducts at industrial scales.

## MATERIALS AND METHODS

### Plasmid construction

All plasmids (containing the pBR322 ori) were prepared by cloning the coding sequences downstream of an arabinose-inducible P_BAD_ promoter using a standard BglBrick expression system as described previously (37, 38). The 5′ ribozyme was added by primer overhang, and the 3′ ribozyme was inserted as a gBlock synthesized from Integrated DNA Technologies, Inc. (Coralville, IA, USA). All protein-coding sequences were codon-optimized for *E. coli* expression (7, 10) and were ligated to the expression vector using NheI/BamHI restriction sites. Proteins used in this work were derived or engineered from *Trichonephila clavipes* Spidroin-1 (accession number: P19837), rabbit soleus titin I-band fragment I65-I70 (accession number: Y14852), *Mytilus galloprovincialis* Mfp5 (accession number: ADQ78274), and human islet amyloid polypeptide (amylin) (accession number: P10997). Each plasmid was separately introduced into *E. coli* NEB10β competent cells to initiate protein production. An additional plasmid carrying the glycine-tRNA gene (glyV) was co-introduced during transformation to enhance the overproduction of high-molecular-weight protein 96× FGAILSS, which is rich in glycine (10, 13).

### GFP production

Fluorescence and cell growth were measured on an Infinite M nano (Tecan, Männedorf, Switzerland) plate reader following previous methods (39, 40). Specifically, cells were grown from single colonies for 8 h in LB media supplemented with 100 µg/mL of ampicillin. Cultures were then diluted to an initial optical density at a wavelength of 600 nm (OD_600_) of 0.01 in LB medium supplemented with ampicillin and 0.03% arabinose and loaded onto a 96-well plate. The 96-well plate was incubated inside the plate reader at 37°C with constant shaking. Cell density (OD_600_) and green fluorescence (excitation: 488 ± 9 nm, emission: 518 ± 20 nm) were recorded every 6 min for 8 h. Background fluorescence from wild-type NEB-10B *E. coli* culture was subtracted from fluorescent measurements. Background-corrected fluorescence was normalized by total cell density in each well using OD_600_ readings. OD_600_ normalized GFP fluorescence at the stationary growth phase (8 h after induction) to compare GFP expression levels.

### Protein production and quantification

A colony of *E. coli* transformed with a plasmid was cultured in LB medium at 37°C on an orbital shaker. The culture was then used to inoculate fresh terrific broth (TB) medium, which was allowed to grow to an OD_600_ of 1–2. The culture was then induced by adding 0.04% arabinose and continued to grow at 30°C overnight. Samples were collected and pelleted, followed by resuspension in Laemmli sample buffer (2% SDS, 10% glycerol, 60 mM Tris at pH 6.8, 0.01% bromophenol blue, 100 µM dithiothreitol [DTT]).

Samples were then loaded onto SDS-PAGE gels, which were 1 mm thick and discontinuous with 5% stacking gel on the top and indicated percentages of separation gels on the bottom. Gels were run on Mini-Protean Tetra Cells (Bio-Rad) in 1× Tris-glycine SDS buffer (25 mM Tris base, 250 mM glycine, 0.1% wt/vol SDS) until just before the dye front exited the gel. The protein expression level was estimated using ImageJ software to quantify the protein band relative to the native 100 kD band for all proteins but Mfp, which was normalized to a smaller protein that was clearer on the higher concentration gel.

### Circular mRNA testing

Circular mRNA was confirmed using a combination of previous protocols (23, 30). Circular GFP expression strains were grown in LB at 37°C, induced at OD_600_ of 0.4, and incubated for 3 h. Total RNA was then extracted using the Zymo Research Quick-RNA Miniprep Plus Kit. The extracted total RNA sample was treated on a column with DNase I to remove any residue in the DNA following the kit protocol. One microgram of purified RNA was later treated with 10 U of Ribonuclease R (Biosearch Technologies) for 30 min at 37°C to digest all linear RNAs. After RNase treatment, samples were reverse transcribed using Thermo Scientific’s RevertAid First Strand cDNA Synthesis Kit following the manufacturer’s protocol for their random primers. The treated samples were then used as PCR templates with forward primers designed to anneal at both ends of GFP to amplify the ligation region. PCR products were run on a 1% agarose gel, and the band was extracted for Sanger sequencing. Sanger sequencing was performed by Azenta Life Sciences (Indianapolis, IN, USA).

To estimate circularization efficiency, 1 µg of total RNA was treated with 1 U of NEB RNase R for 30 min, following the manufacturer’s protocol. The treated RNA was then purified using NEB’s Monarch Spin RNA Cleanup Kit. Following purification, samples were reverse transcribed using Thermo Scientific’s RevertAid First Strand cDNA Synthesis Kit with random primers, according to the manufacturer’s instructions. The resulting cDNA was used as the qPCR template, with primers annealing to the central region of the GFP sequence. RNase R–untreated control samples underwent the same procedure, excluding only the RNase R enzyme, to allow comparison of linear RNA depletion.

## SUPPLEMENTAL MATERIAL

Supplemental material aem.01579-25-s0001.docx Figures S1 to S5; Tables S1 and S2.

ASM does not own the copyrights to Supplemental Material that may be linked to, or accessed through, an article. The authors have granted ASM a non-exclusive, world-wide license to publish the Supplemental Material files. Please contact the corresponding author directly for reuse.
