# Supplementary material for: Enhancing Escherichia coli production of material proteins using circular mRNAs
Source: Appl Environ Microbiol. 2025 Oct 7;91(11):e01579-25. doi: 10.1128/aem.01579-25 (PMC12628768; doi:10.1128/aem.01579-25)
Supplement: Supplemental material — Figures S1 to S5; Tables S1 and S2. [file aem.01579-25-s0001.docx]

**Supplementary Information:**

# **Enhancing *Escherichia coli* production of material proteins using circular mRNAs**

**Authors:** Alden Filko^1^, Fuzhong Zhang^1-3^, *

Affiliations:

^1^Department of Energy, Environmental and Chemical Engineering,

^2^Division of Biological & Biomedical Sciences,

^3^Institute of Materials Science & Engineering,

Washington University in St. Louis, Saint Louis, Missouri, USA.

*e-mail: fzhang@seas.wustl.edu


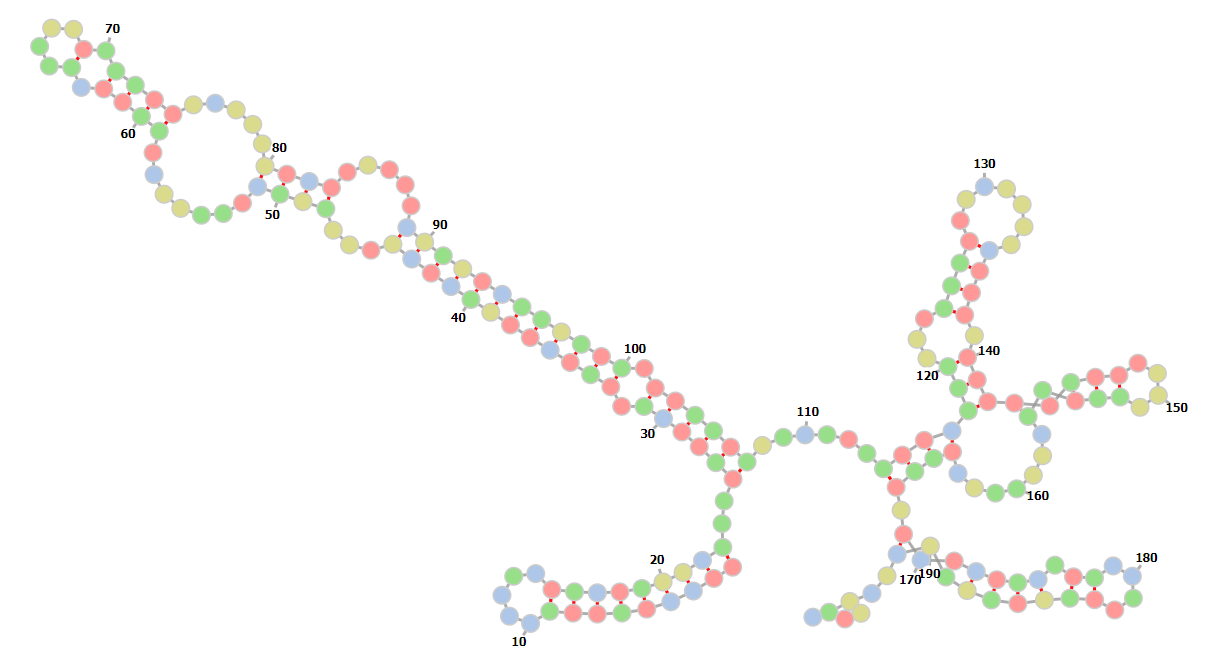


(…GFP…)

**A**

**U**

**G**

**C**

**Supplementary Figure 1**: The predicted secondary structure (by MXfold2^35^) of the circularized region and insulating stem loops of the circular mRNA.


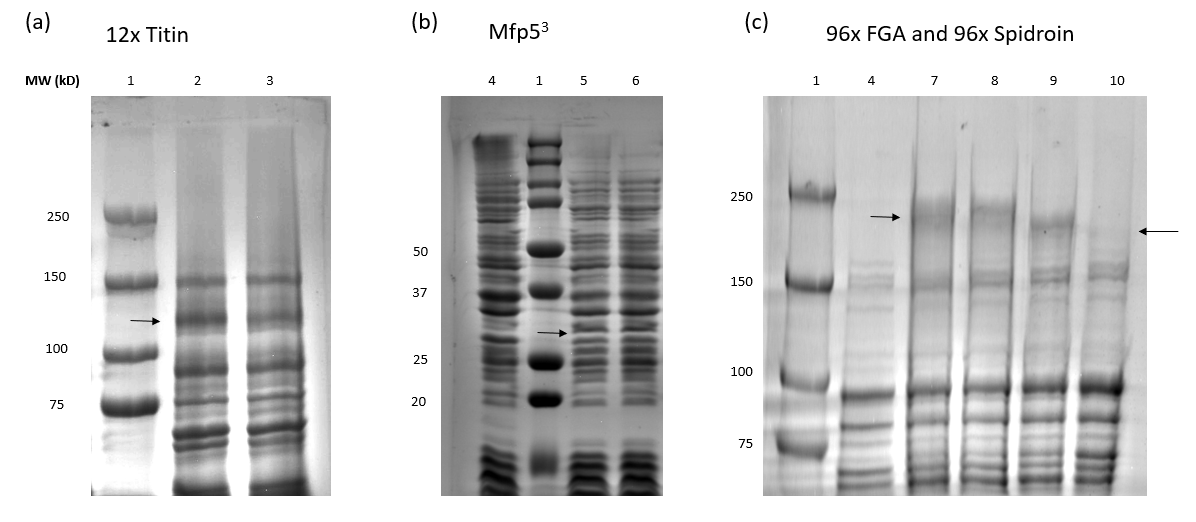


**Supplementary Figure 2.** (a) SDS-PAGE gels showing whole cell lysate of induced 12x Titin production cultures. Lane 1: molecular weight ladder, lane 2: Circ. mRNA 12x Titin, lane 3: Lin. mRNA 12x Titin. The arrow indicates the expected size of the 12x Titin protein. (b) SDS-PAGE gels showing whole cell lysate of induced 3x MFP5^3^ production cultures. Lane 4: WT NEB-10B *E. coli* culture, lane 1: molecular weight ladder, lane 5: Circ. mRNA 3x MFP5^3^, lane 6: Lin. mRNA 3x MFP5^3^. The arrow indicates the expected size of the 3x MFP5^3^ protein. (c) SDS-PAGE gels showing whole cell lysate of induced 96x FGA and 96x spider silk production cultures. Lane 1: molecular weight ladder, lane 4: WT NEB-10B *E. coli* culture, lane 7: Circ. RNA 96x FGA, lane 8: Lin. RNA 96x FGA, lane 9: Circ. RNA 96x spidroin, lane 10: Lin. 96x spidroin. The left and right arrows indicate the expected size of the 96x FGA and 96x Spidroin, respectively.


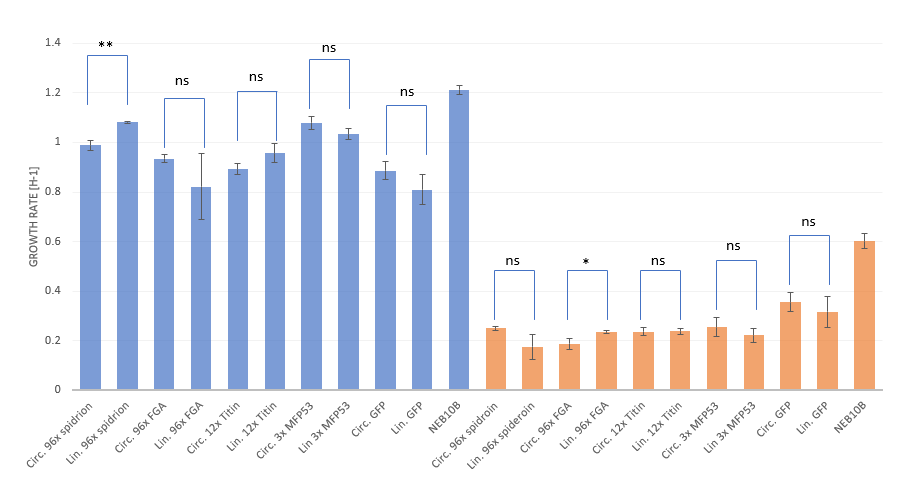


**Supplementary Figure 3.** Growth rates before and after induction of circular and linear mRNA-derived material protein were measured in TB media, with GFP expression assessed in LB media. Cultures were induced in late exponential phase, following the same protocol used for material production. Error bars represent standard deviation from biological replicates (n = 3). * P<0.05, ^ns^ P>0.05,** P<0.005 unpaired t test comparing circular and linear constructs


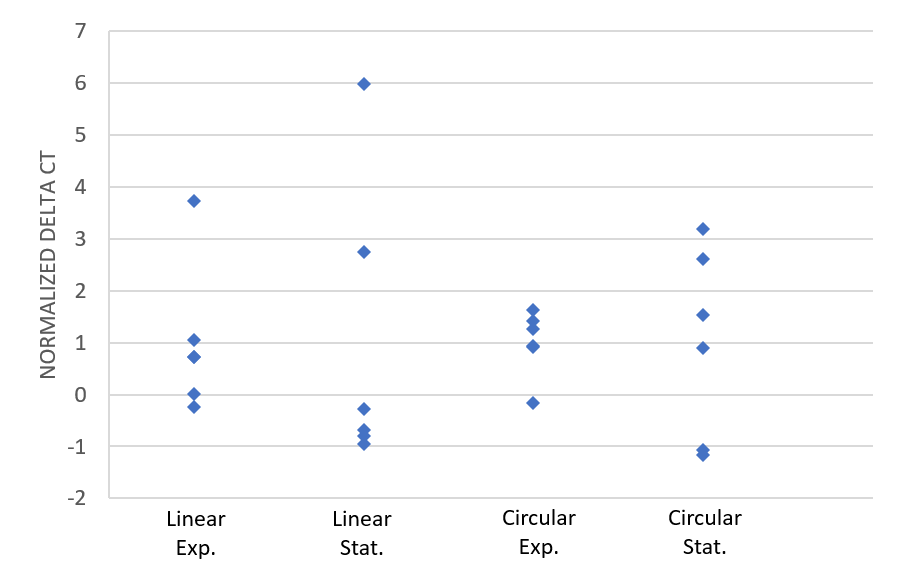


**Supplementary Figure 4:** Normalized mRNA levels of 96x Spidroin in circular or linear mRNA constructs. Primers for 96x Spidroin quantification target the 5’ end of the RNA between the RBS and the first repeat to avoid the primers having multiple binding sites.


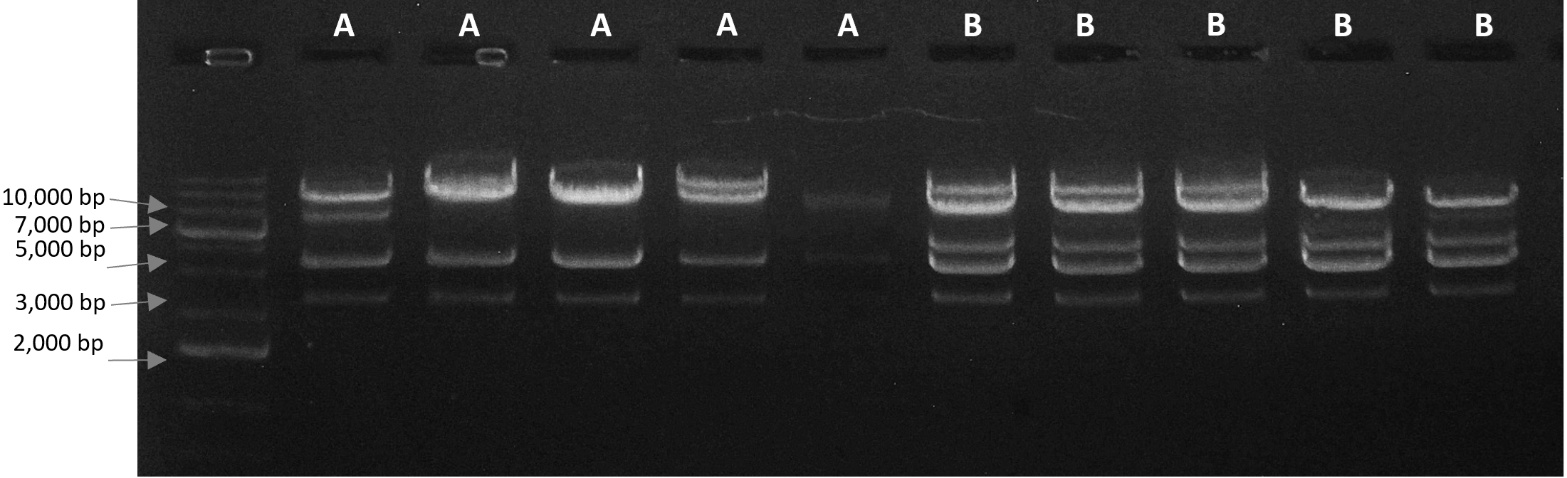


**Supplementary Figure 5.** Stability of expression vectors carrying either the circular (A) or linear (B) 96x spider silk gene. 1% agarose gels showing digested plasmids from individual colonies of 96x spider silk expression plasmid and glyV expression plasmid. The expected constructs should show 10,150bp and 3,500bp bands for spider silk and a 2500bp band from glyV.

**A**

**A**

**A**

**A**

**A**

**B**

**B**

**B**

**B**

**B**

**B**

**B**

**B**

**B**

**B**

**B**

**B**

**B**

**Supplementary Table 1.** DNA sequences of linear and circular GFP with or without the insulation loop. Blue sequences represent the ribozymes, orange sequences represent the stem-loops, and green sequences represent GFP.

| **Construct** | **Sequence** |
| --- | --- |
| Linear GFP | agaattcaaaaGATCTTTTAAGAAGGAGATATACATATGagcaaaggtgaagaactgtttaccggcgttgtgccgattctggtggaactggatggcgatgtgaacggtcacaaattcagcgtgcgtggtgaaggtgaaggcgatgccacgattggcaaactgacgctgaaatttatctgcaccaccggcaaactgccggtgccgtggccgacgctggtgaccaccctgacctatggcgttcagtgttttagtcgctatccggatcacatgaaacgtcacgatttctttaaatctgcaatgccggaaggctatgtgcaggaacgtacgattagctttaaagatgatggcaaatataaaacgcgcgccgttgtgaaatttgaaggcgataccctggtgaaccgcattgaactgaaaggcacggattttaaagaagatggcaatatcctgggccataaactggaatacaactttaatagccataatgtttatattacggcggataaacagaaaaatggcatcaaagcgaattttaccgttcgccataacgttgaagatggcagtgtgcagctggcagatcattatcagcagaataccccgattggtgatggtccggtgctgctgccggataatcattatctgagcacgcagaccgttctgtctaaagatccgaacgaaaaaggcacgcgggaccacatggttctgcacgaatatgtgaatgcggcaggtattacgtggagccatccgcagttcgaaaaataa |
| Circular no loops | aGGGCCGCACTCGCCGGTCCCAAGCCCGGATAAAATGGGAGGGGGCGGGAAACCGCCTAACCATGCCGAGTgaattcaaaaGATCTTTTAAGAAGGAGATATACATATGagcaaaggtgaagaactgtttaccggcgttgtgccgattctggtggaactggatggcgatgtgaacggtcacaaattcagcgtgcgtggtgaaggtgaaggcgatgccacgattggcaaactgacgctgaaatttatctgcaccaccggcaaactgccggtgccgtggccgacgctggtgaccaccctgacctatggcgttcagtgttttagtcgctatccggatcacatgaaacgtcacgatttctttaaatctgcaatgccggaaggctatgtgcaggaacgtacgattagctttaaagatgatggcaaatataaaacgcgcgccgttgtgaaatttgaaggcgataccctggtgaaccgcattgaactgaaaggcacggattttaaagaagatggcaatatcctgggccataaactggaatacaactttaatagccataatgtttatattacggcggataaacagaaaaatggcatcaaagcgaattttaccgttcgccataacgttgaagatggcagtgtgcagctggcagatcattatcagcagaataccccgattggtgatggtccggtgctgctgccggataatcattatctgagcacgcagaccgttctgtctaaagatccgaacgaaaaaggcacgcgggaccacatggttctgcacgaatatgtgaatgcggcaggtattacgtggagccatccgcagttcgaaaaataaGGATCCAAACtcgagCCGCGGTCGGCGTGGACTGTAGAACACTGCCAATGCCGGTCCCAAGCCCGGATAAAAGTGGAGGGTACAGTCCACGC |
| Circular with loops | aGGGCCGCACTCGCCGGTCCCAAGCCCGGATAAAATGGGAGGGGGCGGGAAACCGCCTAACCATGCCGAGTgtgctcgcttcggcagcacatatactaggaattcaaaaGATCTTTTAAGAAGGAGATATACATATGagcaaaggtgaagaactgtttaccggcgttgtgccgattctggtggaactggatggcgatgtgaacggtcacaaattcagcgtgcgtggtgaaggtgaaggcgatgccacgattggcaaactgacgctgaaatttatctgcaccaccggcaaactgccggtgccgtggccgacgctggtgaccaccctgacctatggcgttcagtgttttagtcgctatccggatcacatgaaacgtcacgatttctttaaatctgcaatgccggaaggctatgtgcaggaacgtacgattagctttaaagatgatggcaaatataaaacgcgcgccgttgtgaaatttgaaggcgataccctggtgaaccgcattgaactgaaaggcacggattttaaagaagatggcaatatcctgggccataaactggaatacaactttaatagccataatgtttatattacggcggataaacagaaaaatggcatcaaagcgaattttaccgttcgccataacgttgaagatggcagtgtgcagctggcagatcattatcagcagaataccccgattggtgatggtccggtgctgctgccggataatcattatctgagcacgcagaccgttctgtctaaagatccgaacgaaaaaggcacgcgggaccacatggttctgcacgaatatgtgaatgcggcaggtattacgtggagccatccgcagttcgaaaaataaGGATCCAAACtcgagggttgcggctttctgctgcaatcCCGCGGTCGGCGTGGACTGTAGAACACTGCCAATGCCGGTCCCAAGCCCGGATAAAAGTGGAGGGTACAGTCCACGC |

| 96x spider silk | SGRGGLGGQGAGMAAAAAMGGAGQGGYGGLGSQGTSGRGGLGGQGAGMAAAAAMGGAGQGGYGGLGSQGTSGRGGLGGQGAGMAAAAAMGGAGQGGYGGLGSQGTSGRGGLGGQGAGMAAAAAMGGAGQGGYGGLGSQGTSGRGGLGGQGAGMAAAAAMGGAGQGGYGGLGSQGTSGRGGLGGQGAGMAAAAAMGGAGQGGYGGLGSQGTSGRGGLGGQGAGMAAAAAMGGAGQGGYGGLGSQGTSGRGGLGGQGAGMAAAAAMGGAGQGGYGGLGSQGTSGRGGLGGQGAGMAAAAAMGGAGQGGYGGLGSQGTSGRGGLGGQGAGMAAAAAMGGAGQGGYGGLGSQGTSGRGGLGGQGAGMAAAAAMGGAGQGGYGGLGSQGTSGRGGLGGQGAGMAAAAAMGGAGQGGYGGLGSQGTSGRGGLGGQGAGMAAAAAMGGAGQGGYGGLGSQGTSGRGGLGGQGAGMAAAAAMGGAGQGGYGGLGSQGTSGRGGLGGQGAGMAAAAAMGGAGQGGYGGLGSQGTSGRGGLGGQGAGMAAAAAMGGAGQGGYGGLGSQGTSGRGGLGGQGAGMAAAAAMGGAGQGGYGGLGSQGTSGRGGLGGQGAGMAAAAAMGGAGQGGYGGLGSQGTSGRGGLGGQGAGMAAAAAMGGAGQGGYGGLGSQGTSGRGGLGGQGAGMAAAAAMGGAGQGGYGGLGSQGTSGRGGLGGQGAGMAAAAAMGGAGQGGYGGLGSQGTSGRGGLGGQGAGMAAAAAMGGAGQGGYGGLGSQGTSGRGGLGGQGAGMAAAAAMGGAGQGGYGGLGSQGTSGRGGLGGQGAGMAAAAAMGGAGQGGYGGLGSQGTSGRGGLGGQGAGMAAAAAMGGAGQGGYGGLGSQGTSGRGGLGGQGAGMAAAAAMGGAGQGGYGGLGSQGTSGRGGLGGQGAGMAAAAAMGGAGQGGYGGLGSQGTSGRGGLGGQGAGMAAAAAMGGAGQGGYGGLGSQGTSGRGGLGGQGAGMAAAAAMGGAGQGGYGGLGSQGTSGRGGLGGQGAGMAAAAAMGGAGQGGYGGLGSQGTSGRGGLGGQGAGMAAAAAMGGAGQGGYGGLGSQGTSGRGGLGGQGAGMAAAAAMGGAGQGGYGGLGSQGTSGRGGLGGQGAGMAAAAAMGGAGQGGYGGLGSQGTSGRGGLGGQGAGMAAAAAMGGAGQGGYGGLGSQGTSGRGGLGGQGAGMAAAAAMGGAGQGGYGGLGSQGTSGRGGLGGQGAGMAAAAAMGGAGQGGYGGLGSQGTSGRGGLGGQGAGMAAAAAMGGAGQGGYGGLGSQGTSGRGGLGGQGAGMAAAAAMGGAGQGGYGGLGSQGTSGRGGLGGQGAGMAAAAAMGGAGQGGYGGLGSQGTSGRGGLGGQGAGMAAAAAMGGAGQGGYGGLGSQGTSGRGGLGGQGAGMAAAAAMGGAGQGGYGGLGSQGTSGRGGLGGQGAGMAAAAAMGGAGQGGYGGLGSQGTSGRGGLGGQGAGMAAAAAMGGAGQGGYGGLGSQGTSGRGGLGGQGAGMAAAAAMGGAGQGGYGGLGSQGTSGRGGLGGQGAGMAAAAAMGGAGQGGYGGLGSQGTSGRGGLGGQGAGMAAAAAMGGAGQGGYGGLGSQGTSGRGGLGGQGAGMAAAAAMGGAGQGGYGGLGSQGTSGRGGLGGQGAGMAAAAAMGGAGQGGYGGLGSQGTSGRGGLGGQGAGMAAAAAMGGAGQGGYGGLGSQGTSGRGGLGGQGAGMAAAAAMGGAGQGGYGGLGSQGTSGRGGLGGQGAGMAAAAAMGGAGQGGYGGLGSQGTSGRGGLGGQGAGMAAAAAMGGAGQGGYGGLGSQGTSGRGGLGGQGAGMAAAAAMGGAGQGGYGGLGSQGTSGRGGLGGQGAGMAAAAAMGGAGQGGYGGLGSQGTSGRGGLGGQGAGMAAAAAMGGAGQGGYGGLGSQGTSGRGGLGGQGAGMAAAAAMGGAGQGGYGGLGSQGTSGRGGLGGQGAGMAAAAAMGGAGQGGYGGLGSQGTSGRGGLGGQGAGMAAAAAMGGAGQGGYGGLGSQGTSGRGGLGGQGAGMAAAAAMGGAGQGGYGGLGSQGTSGRGGLGGQGAGMAAAAAMGGAGQGGYGGLGSQGTSGRGGLGGQGAGMAAAAAMGGAGQGGYGGLGSQGTSGRGGLGGQGAGMAAAAAMGGAGQGGYGGLGSQGTSGRGGLGGQGAGMAAAAAMGGAGQGGYGGLGSQGTSGRGGLGGQGAGMAAAAAMGGAGQGGYGGLGSQGTSGRGGLGGQGAGMAAAAAMGGAGQGGYGGLGSQGTSGRGGLGGQGAGMAAAAAMGGAGQGGYGGLGSQGTSGRGGLGGQGAGMAAAAAMGGAGQGGYGGLGSQGTSGRGGLGGQGAGMAAAAAMGGAGQGGYGGLGSQGTSGRGGLGGQGAGMAAAAAMGGAGQGGYGGLGSQGTSGRGGLGGQGAGMAAAAAMGGAGQGGYGGLGSQGTSGRGGLGGQGAGMAAAAAMGGAGQGGYGGLGSQGTSGRGGLGGQGAGMAAAAAMGGAGQGGYGGLGSQGTSGRGGLGGQGAGMAAAAAMGGAGQGGYGGLGSQGTSGRGGLGGQGAGMAAAAAMGGAGQGGYGGLGSQGTSGRGGLGGQGAGMAAAAAMGGAGQGGYGGLGSQGTSGRGGLGGQGAGMAAAAAMGGAGQGGYGGLGSQGTSGRGGLGGQGAGMAAAAAMGGAGQGGYGGLGSQGTSGRGGLGGQGAGMAAAAAMGGAGQGGYGGLGSQGTSGRGGLGGQGAGMAAAAAMGGAGQGGYGGLGSQGTSGRGGLGGQGAGMAAAAAMGGAGQGGYGGLGSQGTSGRGGLGGQGAGMAAAAAMGGAGQGGYGGLGSQGTSGRGGLGGQGAGMAAAAAMGGAGQGGYGGLGSQGTSGRGGLGGQGAGMAAAAAMGGAGQGGYGGLGSQGTSGRGGLGGQGAGMAAAAAMGGAGQGGYGGLGSQGTSGRGGLGGQGAGMAAAAAMGGAGQGGYGGLGSQGTSGRGGLGGQGAGMAAAAAMGGAGQGGYGGLGSQGTSGRGGLGGQGAGMAAAAAMGGAGQGGYGGLGSQGTSGRGGLGGQGAGMAAAAAMGGAGQGGYGGLGSQGTSGRGGLGGQGAGMAAAAAMGGAGQGGYGGLGSQGTSGRGGLGGQGAGMAAAAAMGGAGQGGYGGLGSQGTSGRGGLGGQGAGMAAAAAMGGAGQGGYGGLGSQGTSGRGGLGGQGAGMAAAAAMGGAGQGGYGGLGSQGTSGRGGLGGQGAGMAAAAAMGGAGQGGYGGLGSQGTSGRGGLGGQGAGMAAAAAMGGAGQGGYGGLGSQGTSGRGGLGGQGAGMAAAAAMGGAGQGGYGGLGSQGTSGRGGLGGQGAGMAAAAAMGGAGQGGYGGLGSQGT |
| --- | --- |

**Supplementary Table 2.** Protein sequences of PBMs used in this study.

| 12x titin Ig | PPFFDLKPVSVDLALGESGTFKCHVTGTAPIKITWAKDNREIRPGGNYKMTLVENTATLTVLKVTKGDAGQYTCYASNVAGKDSCSAQLGVQEPPRFIKKLEPSRIVKQDEHTRYECKIGGSPEIKVLWYKDETEIQESSKFRMSFVESVAVLEMYNLSVEDSGDYTCEAHNAAGSASSSTSLKVKEPPVFRKKPHPVETLKGADVHLECELQGTPPFQVSWHKDKRELRSGKKYKIMSENFLTSIHILNVDSADIGEYQCKASNDVGSDTCVGSITLKAPPRFVKKLSDISTVVGEEVQLQATIEGAEPISVAWFKDKGEIVRESDNIWISYSENIATLQFSRAEPANAGKYTCQIKNEAGTQECFATLSVLE |
| --- | --- |
| 96x FGA | GRGGLGGQGAGFGAILSSGGAGQGGYGGLGSQGTSGRGGLGGQGAGFGAILSSGGAGQGGYGGLGSQGTSGRGGLGGQGAGFGAILSSGGAGQGGYGGLGSQGTSGRGGLGGQGAGFGAILSSGGAGQGGYGGLGSQGTSGRGGLGGQGAGFGAILSSGGAGQGGYGGLGSQGTSGRGGLGGQGAGFGAILSSGGAGQGGYGGLGSQGTSGRGGLGGQGAGFGAILSSGGAGQGGYGGLGSQGTSGRGGLGGQGAGFGAILSSGGAGQGGYGGLGSQGTSGRGGLGGQGAGFGAILSSGGAGQGGYGGLGSQGTSGRGGLGGQGAGFGAILSSGGAGQGGYGGLGSQGTSGRGGLGGQGAGFGAILSSGGAGQGGYGGLGSQGTSGRGGLGGQGAGFGAILSSGGAGQGGYGGLGSQGTSGRGGLGGQGAGFGAILSSGGAGQGGYGGLGSQGTSGRGGLGGQGAGFGAILSSGGAGQGGYGGLGSQGTSGRGGLGGQGAGFGAILSSGGAGQGGYGGLGSQGTSGRGGLGGQGAGFGAILSSGGAGQGGYGGLGSQGTSGRGGLGGQGAGFGAILSSGGAGQGGYGGLGSQGTSGRGGLGGQGAGFGAILSSGGAGQGGYGGLGSQGTSGRGGLGGQGAGFGAILSSGGAGQGGYGGLGSQGTSGRGGLGGQGAGFGAILSSGGAGQGGYGGLGSQGTSGRGGLGGQGAGFGAILSSGGAGQGGYGGLGSQGTSGRGGLGGQGAGFGAILSSGGAGQGGYGGLGSQGTSGRGGLGGQGAGFGAILSSGGAGQGGYGGLGSQGTSGRGGLGGQGAGFGAILSSGGAGQGGYGGLGSQGTSGRGGLGGQGAGFGAILSSGGAGQGGYGGLGSQGTSGRGGLGGQGAGFGAILSSGGAGQGGYGGLGSQGTSGRGGLGGQGAGFGAILSSGGAGQGGYGGLGSQGTSGRGGLGGQGAGFGAILSSGGAGQGGYGGLGSQGTSGRGGLGGQGAGFGAILSSGGAGQGGYGGLGSQGTSGRGGLGGQGAGFGAILSSGGAGQGGYGGLGSQGTSGRGGLGGQGAGFGAILSSGGAGQGGYGGLGSQGTSGRGGLGGQGAGFGAILSSGGAGQGGYGGLGSQGTSGRGGLGGQGAGFGAILSSGGAGQGGYGGLGSQGTSGRGGLGGQGAGFGAILSSGGAGQGGYGGLGSQGTSGRGGLGGQGAGFGAILSSGGAGQGGYGGLGSQGTSGRGGLGGQGAGFGAILSSGGAGQGGYGGLGSQGTSGRGGLGGQGAGFGAILSSGGAGQGGYGGLGSQGTSGRGGLGGQGAGFGAILSSGGAGQGGYGGLGSQGTSGRGGLGGQGAGFGAILSSGGAGQGGYGGLGSQGTSGRGGLGGQGAGFGAILSSGGAGQGGYGGLGSQGTSGRGGLGGQGAGFGAILSSGGAGQGGYGGLGSQGTSGRGGLGGQGAGFGAILSSGGAGQGGYGGLGSQGTSGRGGLGGQGAGFGAILSSGGAGQGGYGGLGSQGTSGRGGLGGQGAGFGAILSSGGAGQGGYGGLGSQGTSGRGGLGGQGAGFGAILSSGGAGQGGYGGLGSQGTSGRGGLGGQGAGFGAILSSGGAGQGGYGGLGSQGTSGRGGLGGQGAGFGAILSSGGAGQGGYGGLGSQGTSGRGGLGGQGAGFGAILSSGGAGQGGYGGLGSQGTSGRGGLGGQGAGFGAILSSGGAGQGGYGGLGSQGTSGRGGLGGQGAGFGAILSSGGAGQGGYGGLGSQGTSGRGGLGGQGAGFGAILSSGGAGQGGYGGLGSQGTSGRGGLGGQGAGFGAILSSGGAGQGGYGGLGSQGTSGRGGLGGQGAGFGAILSSGGAGQGGYGGLGSQGTSGRGGLGGQGAGFGAILSSGGAGQGGYGGLGSQGTSGRGGLGGQGAGFGAILSSGGAGQGGYGGLGSQGTSGRGGLGGQGAGFGAILSSGGAGQGGYGGLGSQGTSGRGGLGGQGAGFGAILSSGGAGQGGYGGLGSQGTSGRGGLGGQGAGFGAILSSGGAGQGGYGGLGSQGTSGRGGLGGQGAGFGAILSSGGAGQGGYGGLGSQGTSGRGGLGGQGAGFGAILSSGGAGQGGYGGLGSQGTSGRGGLGGQGAGFGAILSSGGAGQGGYGGLGSQGTSGRGGLGGQGAGFGAILSSGGAGQGGYGGLGSQGTSGRGGLGGQGAGFGAILSSGGAGQGGYGGLGSQGTSGRGGLGGQGAGFGAILSSGGAGQGGYGGLGSQGTSGRGGLGGQGAGFGAILSSGGAGQGGYGGLGSQGTSGRGGLGGQGAGFGAILSSGGAGQGGYGGLGSQGTSGRGGLGGQGAGFGAILSSGGAGQGGYGGLGSQGTSGRGGLGGQGAGFGAILSSGGAGQGGYGGLGSQGTSGRGGLGGQGAGFGAILSSGGAGQGGYGGLGSQGTSGRGGLGGQGAGFGAILSSGGAGQGGYGGLGSQGTSGRGGLGGQGAGFGAILSSGGAGQGGYGGLGSQGTSGRGGLGGQGAGFGAILSSGGAGQGGYGGLGSQGTSGRGGLGGQGAGFGAILSSGGAGQGGYGGLGSQGTSGRGGLGGQGAGFGAILSSGGAGQGGYGGLGSQGTSGRGGLGGQGAGFGAILSSGGAGQGGYGGLGSQGTSGRGGLGGQGAGFGAILSSGGAGQGGYGGLGSQGTSGRGGLGGQGAGFGAILSSGGAGQGGYGGLGSQGTSGRGGLGGQGAGFGAILSSGGAGQGGYGGLGSQGTSGRGGLGGQGAGFGAILSSGGAGQGGYGGLGSQGTSGRGGLGGQGAGFGAILSSGGAGQGGYGGLGSQGTSGRGGLGGQGAGFGAILSSGGAGQGGYGGLGSQGTSGRGGLGGQGAGFGAILSSGGAGQGGYGGLGSQGTSGRGGLGGQGAGFGAILSSGGAGQGGYGGLGSQGTSGRGGLGGQGAGFGAILSSGGAGQGGYGGLGSQGTSGRGGLGGQGAGFGAILSSGGAGQGGYGGLGSQGTSGRGGLGGQGAGFGAILSSGGAGQGGYGGLGSQGTSGRGGLGGQGAGFGAILSSGGAGQGGYGGLGSQGTSGRGGLGGQGAGFGAILSSGGAGQGGYGGLGSQGTSGRGGLGGQGAGFGAILSSGGAGQGGYGGLGSQGTSGRGGLGGQGAGFGAILSSGGAGQGGYGGLGSQGTSGRGGLGGQGAGFGAILSSGGAGQGGYGGLGSQGTSGRGGLGGQGAGFGAILSSGGAGQGGYGGLGSQGTSGRGGLGGQGAGFGAILSSGGAGQGGYGGLGSQGTSGRGGLGGQGAGFGAILSSGGAGQGGYGGLGSQGTSGRGGLGGQGAGFGAILSSGGAGQGGYGGLGSQGTSGRGGLGGQGAGFGAILSSGGAGQGGYGGLGSQG |
| 3x MFP | SSEEYKGGYYPGNTYHYHSGGSYHGSGYHGGYKGKYYGKAKKYYYKYKNSGKYKYLKKARKYHRKGYKKYYGGGSSSSEEYKGGYYPGNTYHYHSGGSYHGSGYHGGYKGKYYGKAKKYYYKYKNSGKYKYLKKARKYHRKGYKKYYGGGSSSSEEYKGGYYPGNTYHYHSGGSYHGSGYHGGYKGKYYGKAKKYYYKYKNSGKYKYLKKARKYHRKGYKKYYGGGSS |
